# A shared transcriptional program in early breast neoplasias despite genetic and clinical distinctions

**DOI:** 10.1186/gb-2014-15-5-r71

**Published:** 2014-05-23

**Authors:** Alayne L Brunner, Jun Li, Xiangqian Guo, Robert T Sweeney, Sushama Varma, Shirley X Zhu, Rui Li, Robert Tibshirani, Robert B West

**Affiliations:** 1 Department of Pathology, Stanford University School of Medicine, 269 Campus Drive, Stanford, CA 94305-5324, USA; 2 Department of Applied and Computational Mathematics and Statistics, University of Notre Dame, 153 Hurley Hall, Notre Dame, IN 46556, USA; 3 Department of Statistics, Stanford University, 390 Serra Mall, Stanford, CA 94305-4065, USA; 4 Department of Health Research and Policy, Stanford University School of Medicine, 269 Campus Drive, Stanford, CA 94305-5405, USA

## Abstract

**Background:**

The earliest recognizable stages of breast neoplasia are lesions that represent a heterogeneous collection of epithelial proliferations currently classified based on morphology. Their role in the development of breast cancer is not well understood but insight into the critical events at this early stage will improve efforts in breast cancer detection and prevention. These microscopic lesions are technically difficult to study so very little is known about their molecular alterations.

**Results:**

To characterize the transcriptional changes of early breast neoplasia, we sequenced 3′- end enriched RNAseq libraries from formalin-fixed paraffin-embedded tissue of early neoplasia samples and matched normal breast and carcinoma samples from 25 patients. We find that gene expression patterns within early neoplasias are distinct from both normal and breast cancer patterns and identify a pattern of pro-oncogenic changes, including elevated transcription of *ERBB2*, *FOXA1*, and *GATA3* at this early stage. We validate these findings on a second independent gene expression profile data set generated by whole transcriptome sequencing. Measurements of protein expression by immunohistochemistry on an independent set of early neoplasias confirms that ER pathway regulators FOXA1 and GATA3, as well as ER itself, are consistently upregulated at this early stage. The early neoplasia samples also demonstrate coordinated changes in long non-coding RNA expression and microenvironment stromal gene expression patterns.

**Conclusions:**

This study is the first examination of global gene expression in early breast neoplasia, and the genes identified here represent candidate participants in the earliest molecular events in the development of breast cancer.

## Background

Recent large genomic studies have identified and confirmed numerous recurrent mutations and aneuploidies that stratify breast carcinomas across clinicopathologic features [1,2]. However, the events involved early in the evolution of normal breast tissue into invasive breast cancer are still poorly understood. Understanding the initiating driver events in breast cancer progression is a key goal in breast cancer research as it can lead to improvements in early-stage diagnosis, treatment, and prevention strategies [3].

**Figure 1:**
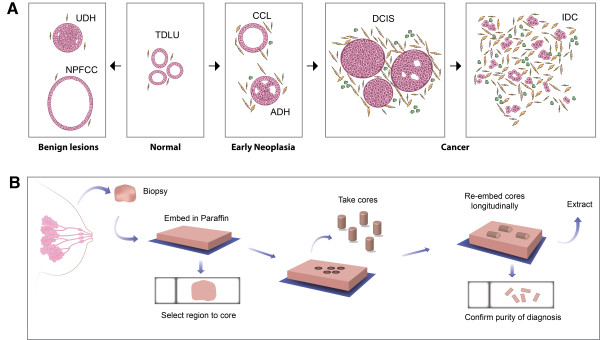
**Early neoplasias in histological specimens. (A)** Schematic of the morphology of early neoplasias, including columnar cell lesions (CCL) and atypical ductal hyperplasia (ADH), as well as later stage ductal carcinoma *in situ* (DCIS), and invasive ductal carcinoma (IDC). Usual ductal hyperplasia (UDH) and non-proliferative fibrocystic change (NPFCC) present benign hyperplasias of the breast. Normal breast is shown as terminal ductal lobular units (TDLU). **(B)** Workflow diagram for lesion purification and RNA isolation.

While the molecular steps required for progression to invasive carcinoma are currently unclear, morphologic studies of breast biopsy tissue [4-6] suggest early neoplasias such as flat epithelial atypia, atypical ductal hyperplasia (ADH), and possibly columnar cell lesion, represent direct precursors to ductal carcinoma *in situ* (DCIS), itself a precursor for invasive ductal carcinoma (IDC) (Figure 1A). It has been shown that the presence of early neoplasias in breast biopsies increases the risk for breast cancer. However, predicting which patients with neoplasia will progress to invasive cancer remains difficult. Pre-invasive lesions diagnosed as ADH and DCIS are associated with progression to invasive cancer in only a fraction of patients: 20% of ADH will be associated with IDC [5] and 50% of DCIS will progress to IDC [6]. This clinical heterogeneity makes treatment of patients with early neoplasia problematic and motivates research aimed at uncovering the molecular mechanisms at play in these earliest stages of cancer development.

We recently performed the first whole genome sequencing of early neoplasias to examine the molecular changes during breast cancer evolution [7]. We found that early neoplasias have already acquired a significant number of genomic alterations: many of the early neoplasias studied possess hundreds of single nucleotide mutations and several chromosome aneuploidies. Many of these alterations are observed in both the patient’s early neoplasia and associated invasive cancer in a significant fraction of instances (4 of 6 sequenced patients, 4 of 14 early neoplasias). These findings indicate that a common ancestral clone develops mutations at a very early stage, before giving rise to both the early neoplasia and related cancer. While most of the single nucleotide variations were not shared between patients, gain of chromosome 1q and activating mutations in *PIK3CA* were observed recurrently in some of the early neoplasia samples. A previous targeted study of cancer hotspot mutations also identified *PIK3CA* as a common mutation present in roughly half of early neoplasias, but not necessarily correlated with progression to invasive carcinoma [8]. These findings highlight the genetic heterogeneity among early neoplasias, consistent with the observed clinical heterogeneity in outcomes. Given a shared origin for early neoplasias and the adjacent invasive cancer, the early neoplasia mutations identified thus far represent molecular events that may be important at this very early stage and suggest that further characterization of early neoplasias represents a unique and promising tool for uncovering additional alterations and elucidating the molecular mechanisms necessary for cancer initiation.

Gene expression has been a particularly effective tool for classifying invasive breast cancer [9], perhaps because it provides a downstream readout that reflects the combination of genetic and epigenetic alterations present in cells. To better understand the molecular events characterizing early neoplasia as a whole, we assessed the transcriptional changes present within a collection of early neoplasias from either patients possessing adjacent cancer or patients with no concurrent cancer.

## Results

### 3SEQ gene expression profiling for early neoplasias, normal tissue, and adjacent cancer

To evaluate gene expression in early neoplasias and allow for comparison with patient-matched normal and cancer samples, we studied archival samples of normal breast, early neoplasia, carcinoma *in situ*, and invasive carcinoma from breast resection specimens (Figure 1A) [7,8]. The samples of carcinoma *in situ*, and invasive carcinoma represent a spectrum of grade and molecular subclass (additional details are included in Table S1 in Additional file 1). To ensure the purity of the cells within the samples, 2-mm diameter tissue cores were re-embedded in paraffin on their sides, re-sectioned, and stained to allow longitudinal examination of the cells across the depth of the tissue cores (Figure 1B). Only samples that possessed >90% of luminal cells with the appropriate diagnosis were included among our samples.

We employed 3SEQ (3′-end sequencing for expression quantification) to quantify RNA from the formalin-fixed paraffin-embedded (FFPE) tissue core specimens. Previously, we demonstrated the feasibility and utility of applying this 3′-end sequence tag counting method on RNA from FFPE material to obtain quantitative global gene expression data for both known mRNAs [10] and long non-coding RNAs (lncRNAs) [11] and to discover novel transcripts [11]. Here we collected RNA from 72 samples (24 normal, 25 early neoplasia, 9 DCIS and 14 IDC) from a total of 25 patients to study a collection of early neoplasias and enable comparisons across a panel of matched patient samples. For 16 patients, complete sets of matched normal, early neoplasia, and cancer (either DCIS or IDC) were present. For six of the cases, concurrent cancer (DCIS or IDC) was not present. 3SEQ libraries were prepared from this collection of RNA samples and directional next-generation sequencing of these libraries yielded an average of 7 million uniquely mapping reads per sample (Table S2 in Additional file 1). Reads mapping to RefSeq genes (n = 22,775) and lncRNAs (n = 2,136) were counted for use in subsequent analysis (see Materials and methods).

We generated a second dataset of gene expression profiles for validation using an independent set of patient-matched breast neoplasia progression samples, including normal, early neoplasia, and IDC. Samples were obtained using methods identical to the original cohort. However, for this validation cohort we used full transcriptome RNAseq, not 3SEQ, with rRNA depleted non-polyA selected libraries. The new RNAseq dataset contains 42 samples: 8 normal, 16 early neoplasia, and 18 cancer (IDC and DCIS) samples. The average mapped reads per sample was 61 million reads.

### Gene expression profiles define an early neoplasia transcriptional program

**Figure 2:**
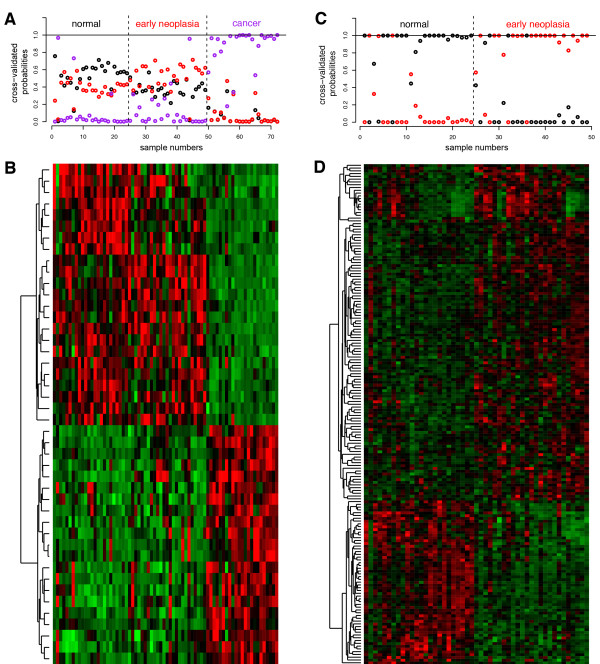
**Classification analyses show early neoplasia is distinct from normal tissue and cancer. (A)** Cross-validated probabilities from the unpaired three-class PAM analysis predicting the diagnostic class of each sample. Classes are normal, early neoplasia, and cancer (includes DCIS and IDC). Samples are arranged as in Table S3 in Additional file 1. The pathologist-assigned diagnosis is listed above. The circles represent the predictions based on the gene expression of the 44 selected genes. The probabilities for each sample sum to 1. **(B)** Mean-centered, normalized heatmap showing 3SEQ transcript levels for each of the 44 genes selected in the three-class PAM analysis in **(A)**. Samples are arranged as in **(A)**. Red is high expression; green is low expression. **(C)** Cross-validated probabilities from the unpaired two-class (normal versus early neoplasia) PAM analysis for predicting the diagnostic class of each sample. Normal and early neoplasia samples are arranged as in Table S3 in Additional file 1. The pathologist-assigned diagnosis is listed above. The circles represent the predictions based on the gene expression of the 180 selected genes. The probabilities for each sample sum to 1. **(D)** Mean-centered, normalized heatmap showing 3SEQ transcript levels for each of the 180 genes selected in the two-class PAM analysis in **(C)**. Samples are arranged as in **(C)**. Red is high expression; green is low expression.

With the gene expression data from the patient-matched early neoplasias, normal breast tissues, and breast cancers, we characterized early neoplasia gene expression and compared the early neoplasias profiles to the normal and cancer samples. A three-class Prediction Analysis of Microarrays (PAM) classification analysis tailored to sequencing data (see Materials and methods) showed that normal, early neoplasia, and cancer samples can be correctly classified into their respective groups using only 44 genes, suggesting that these diagnoses represent distinct groups of samples that can be characterized using strong patterns of shared gene expression (unpaired three-class analysis; cross-validated misclassification rate = 16.7%; Figure 2A,B; Tables S3 and S4 in Additional file 1; see Materials and methods for details). These 44 classification genes are representative of the global expression patterns for these samples, which show large expression differences common among the cancer samples relative to normal tissue and early neoplasia.

We find similar results when excluding the DCIS cases in our analysis and only comparing the normal and early neoplasia samples to IDC. When classification is performed by PAMR [12] using only the 13 invasive cancer samples, along with 24 normal samples and 25 early neoplasia samples we observe a cross-validation misclassification rate of 15.9% using 158 genes. Again gene expression of a relatively small number of genes appears sufficient to distinguish these entities. We used publicly available data from The Cancer Genome Atlas (TCGA) project to validate our findings of our 44 classification genes. TCGA has made available RNAseq data from breast invasive carcinoma samples and the matched normal samples. We extracted the data for 20,502 genes in 45 breast invasive carcinoma samples and 16 matched normal samples, filtering and excluding 2,212 genes with insufficient numbers of reads across samples. After applying SAMseq to detect significant genes and estimate the adjusted *P*-values (false discovery rate (FDR)), we find that in this TCGA data nearly all of the 44 genes are significant between the cancer and normal samples. Only two of the 44 significant genes (PMEPA1 and CCL19) from our cancer/neoplasia/normal classification analysis are not significant in the TCGA dataset (adjusted *P*-values >0.05). Of the 42 significant genes, 33 are extremely significant (adjusted *P*-values <1E-5). These results validate 3SEQ as a quantitative method for analyzing breast cancer samples.

When SAMseq was performed to characterize more completely the differentially expressed genes between the various groups in our 3SEQ dataset, we obtained 3,197 genes that were differentially expressed in cancer versus normal (1,587 up-regulated and 1,610 down-regulated) and 2,597 genes showed differences between cancer and early neoplasia (1,276 up-regulated and 1,321 down-regulated; two-class paired SAMseq [13]; Table 1; Tables S5 and S6 in Additional file 1; see Materials and methods).

**Table 1:** RefSeq genes differentially expressed

	**Genes up**	**Genes down**	**Total genes differentially expressed**
Normal versus early neoplasia	565 (4.1%)	190 (1.4%)	755 (5.5%)
Normal versus cancer	1,587 (11.6%)	1,610 (11.8%)	3,197 (23.4%)
Early neoplasia versus cancer	1,276 (9.4%)	1,321 (9.7%)	2,597 (19.0%)

Paired SAMseq analysis (n = 13,643), FDR <5%. Sixteen samples per class. Numbers and percentages reported are genes up- or down-regulated in early neoplasia relative to normal, and cancer relative to either normal tissue or early neoplasia, respectively.

In a comparison with our validation dataset, these findings are supported as we find 1,332 (42%) of the genes defining cancer versus early neoplasia are significantly differentially expressed (*P*-value <0.05, *t*-test) in the RNAseq data. Furthermore, we find that among the 1,332 genes that are significant in both datasets, 1,323 (99%) agree in the direction of change in expression (Table S14 in Additional file 1).

The normal and early neoplasia samples display more similar global patterns of gene expression compared to the cancer samples, and in a three-way comparison with cancer, the differences between the normal and early neoplasia samples are obscured. To better characterize the differences distinguishing early neoplasia from normal, we performed a second PAMR classification analysis using only normal and early neoplasia samples to determine the genes needed to classify these samples into their respective groups. A minimum of 180 genes is required to correctly classify 85.7% of the normal and early neoplasia samples (unpaired two-class analysis; cross-validated misclassification rate = 14.3%; Figure 2C,D; Tables S3 and S4 in Additional file 1). When all genes differentially expressed between these two groups were identified, 565 genes were up-regulated and 190 genes were down-regulated in the early neoplasias relative to normal (two-class paired SAMseq; Table 1; Table S7 in Additional file 1; see Materials and methods). Of these genes, 76% agree in the direction of expression change in the early neoplasia versus normal analysis comparison in the validation data set (Table S15 in Additional file 1). These genes represent the set of transcriptional changes occurring recurrently across many patients as these lesions develop from normal breast to precursors of cancer.

### Early neoplasias express breast cancer genes

Thousands of genes have been associated with breast cancer, but which of these are crucial for the early stages of cancer development remains uncertain. Those gene events shared by both breast cancer and early neoplasias represent a unique panel of candidates by which to explore potential important events during the earliest stages of carcinogenesis. We first compared those genes differing between early neoplasias and normal breast (n = 755; Table S7 in Additional file 1) with those genes identified as significantly altered between cancer and normal in our dataset (n = 3,197; Table S5 in Additional file 1). Over half of the neoplasia genes (456/755; 60%) overlapped the breast cancer gene list, and most of these genes showed expression changes in the same direction as the cancer genes relative to normal (310 genes up-regulated and 126 genes down-regulated in both neoplasia and cancer relative to normal). The list of genes up-regulated in both neoplasia and breast cancer includes well-known Cancer Gene Census genes [14] such as *ERRB2*, *GATA3*, and *MUC1*, among others (Table S7 in Additional file 1). In fact, genes differentially altered in early neoplasias were enriched for breast cancer genes, including those genes comprising the intrinsic (21 of 306 genes; *P* = 3.3 × 10^-3^; hypergeometric test) [15] and PAM50 (9 of 50 genes; *P* = 7.2 × 10^-5^; hypergeometric test) [16] gene signatures for breast cancer subtypes as well as genes included in the Genes-to-Systems Breast Cancer Database (151 of 2,278; *P* = 9.8 × 10^-13^; hypergeometric test) (Table S7 in Additional file 1) [17].

Observing cancer gene alterations within early neoplasias indicates that these genes may be important for establishing some of the earliest changes necessary to transform normal cells into pre-cancerous cells. Master regulators active at this early stage are of particular interest because of their power to affect entire transcriptional programs. Elevated levels of the proto-oncogene *ERRB2*, which encodes the HER2/neu protein, are often found in the context of amplified gene copy number in HER2+ breast cancers and are important for activating a number of signal transduction pathways and driving cancer progression [18]. While only three of the breast cancers in this study are HER2+ (as classified by clinical pathology immunohistochemistry (IHC) stain; Table S1 in Additional file 1), *ERRB2* transcript levels were elevated in several of the early neoplasia samples relative to normal and suggests a possible function for HER2 at this early stage.

Early neoplasias are most often associated with estrogen receptor-positive (ER+), luminal-type breast cancers, so events present in the early neoplasias may highlight genes important within the luminal cancer development pathway. Notably, altered gene expression was observed in early neoplasias for three of the 57 genes identified as commonly mutated in breast cancer by TCGA project [1]. These genes, *FOXA1*, *GATA3*, and *MYB*, all showed greater mutation rates in ER+/luminal cancers relative to other breast cancer subtypes and, here, possessed up-regulated expression in early neoplasias relative to normal. These genes, therefore, may play a specific and important role in the early stages of ER+/luminal breast cancer development. *PIK3CA*, on the other hand, is commonly mutated in both early neoplasias (approximately 50%) [8] and ER+/luminal breast cancers (approximately 20%) [1] but does not show significant expression changes in the early neoplasias. Additionally, when early neoplasias with a *PIK3CA* mutation were compared to early neoplasias without an identifiable *PIK3CA* mutation (Table S1 in Additional file 1), no significant gene expression differences were identified between the two groups (data not shown). This suggests that while *PIK3CA* activating mutations may be important for generating early neoplasia, they may not be a prominent player in promoting the progression to cancer at this early stage, despite known *PIK3CA*-associated expression differences at the carcinoma stage [19].

Gain of a copy of chromosome 1q is also a common event observed in breast cancers and this gain was previously identified in 4 of 14 sequenced early neoplasias [7]. Fluorescence *in situ* hybridization (FISH) was performed on 10 early neoplasias in this study for which tissue was available. Two of ten early neoplasias showed a copy number gain of chromosome 1q when compared with chromosomes 2q and 3q (Table S8 in Additional file 1), yet amplified samples (n = 2) and wild-type samples (n = 8) were indistinguishable using gene expression classification with PAM (data not shown). Interestingly, 98 genes from across the genome were down-regulated in the samples with the 1q gain compared to the wild-type samples (Table S9 in Additional file 1), suggesting this recurrent genetic event may cause global expression differences within the early neoplasias.

Gene ontology analysis identified several pathways that do not involve known breast oncogenes that are enriched in early neoplasia versus normal (Table S12 in Additional file 1). These include pathways that influence membrane transport, including endocytosis ABC transporters.

### Elevated estrogen receptor pathway genes in most early neoplasias

Despite genetic heterogeneity of the early neoplasias in this study (chromosome 1q and *PIK3CA*), a shared program of transcriptional modifications appears to characterize early neoplasias as a whole. Given the association of early neoplasias with ER + breast cancers, we wished to further characterize two important transcription factors, FOXA1 and GATA3, thought to act upstream of ER in the estrogen response pathway. Both genes showed elevated transcript levels in both the early neoplasia and cancer samples. In fact, *FOXA1* and *GATA3* showed expression levels up-regulated at least 50% in 19 (86%) and 16 (73%) of 22 patients, respectively, when individual matched normal and neoplasia levels were compared (Table S7 in Additional file 1).

**Figure 3:**
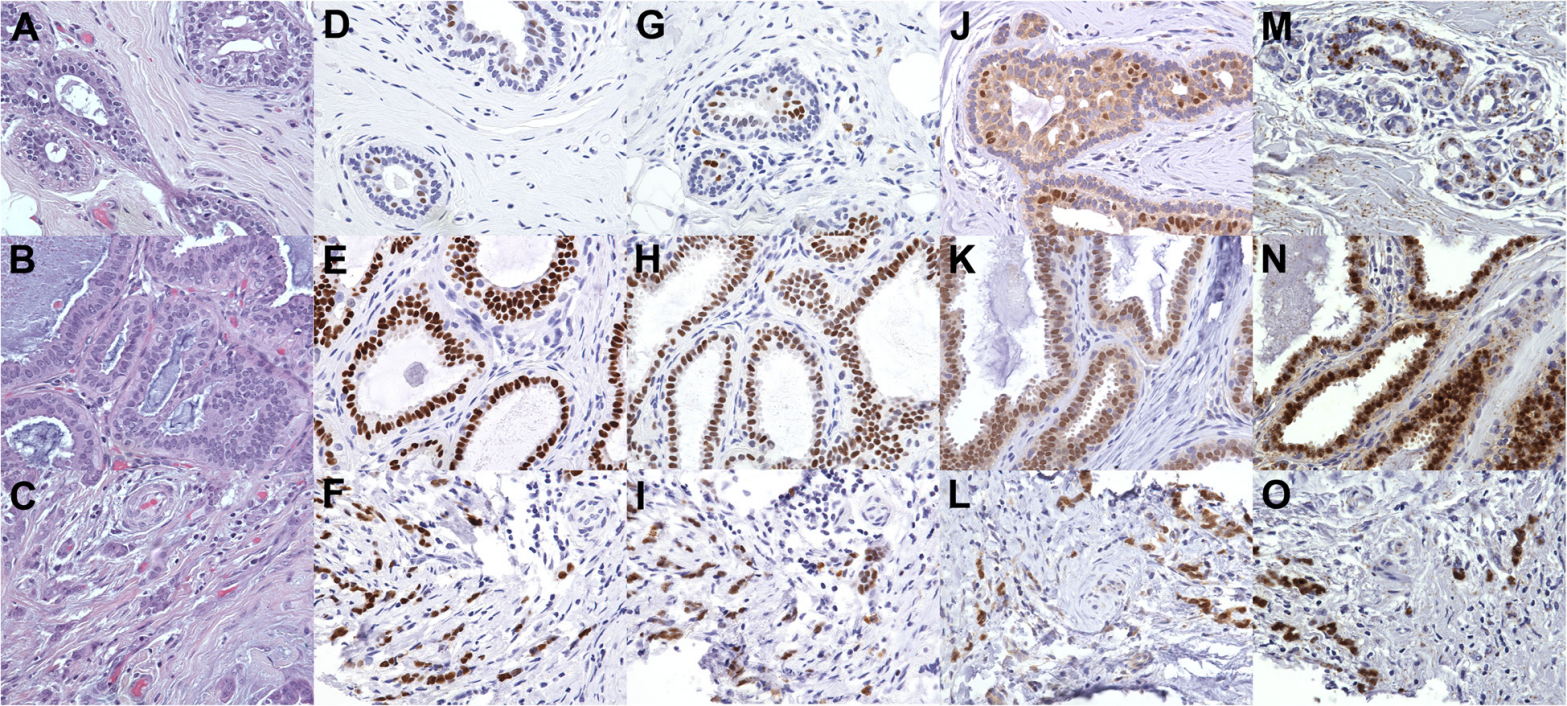
**ER, GATA3, FOXA1, and transcript 13741 are up-regulated in early neoplasia and cancer.** Images of IHC for ER, GATA3, and FOXA1 for a matched set of normal breast, early neoplasia, and invasive ductal carcinoma (donor ID 22666) at 400×. **(A)** Hematoxylin and eosin (H&E) staining of normal tissue; **(B)** H&E of early neoplasia; **(C)** H&E of invasive ductal carcinoma; **(D)** ER in normal tissue; **(E)** ER in early neoplasia; **(F)** ER in invasive ductal carcinoma; **(G)** GATA3 in normal tissue; **(H)** GATA3 in early neoplasia; **(I)** GATA3 in invasive ductal carcinoma; **(J)** FOXA1 in normal tissue; **(K)** FOXA1 in early neoplasia; **(L)** FOXA1 in invasive ductal carcinoma **(M-O)** RNA *in situ* hybridization for transcript 13741 in normal tissue **(M)**, early neoplasia **(N)**, and invasive ductal carcinoma **(O)**.

**Figure 4:**
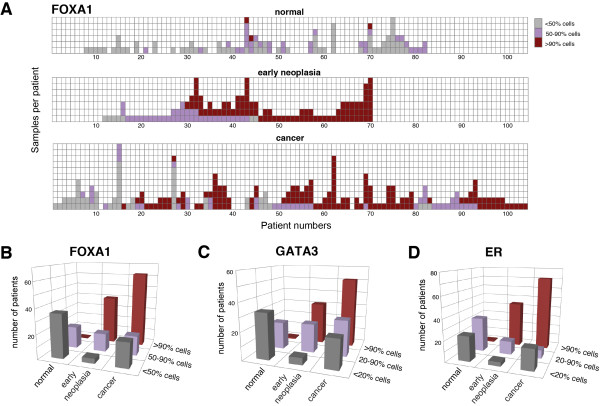
**ER, FOXA1, and GATA3 are elevated in most early neoplasias and cancer. (A)** Visualization of FOXA1 IHC scores for each patient and diagnosis. Each scored sample (tissue core) is a colored box. When multiple samples were scored for a given patient, the boxes are stacked. Patients are roughly sorted by early neoplasia scores. Patients are ordered the same for normal, early neoplasia, and cancer samples, so columns are aligned for patient-matched comparisons between diagnoses. Median IHC scores were used to obtain a single score for each diagnosis per patient. **(B-D)** These patient scores are plotted for FOXA1 **(B)**, GATA3 **(C)**, and ER **(D)**.

To examine the expression of these potential master regulators in an independent collection of early neoplasia cases, we performed IHC on a large number of samples from 104 patients. Six tissue microarrays possessing various combinations of normal breast, early neoplasia, DCIS, and IDC within 534 tissue cores were constructed for this study. These tissue microarrays were stained for ER, FOXA1, and GATA3, as well as with hematoxylin and eosin (Figure 3A-C) to confirm the diagnoses within each tissue core. Cores were scored for protein expression by estimating the fraction of cells staining positive for each tissue core, creating a dataset with multiple samples per diagnosis for several of the patients (Figure 4A; Table S10 in Additional file 1). Multiple samples from the same patient were sometimes heterogeneous in staining (patient 45; Figure 4A) and sometimes remarkably similar with a given diagnosis (patient 62; Figure 4A).

Strikingly, and as a validation for the ability of 3SEQ to identify expression differences of luminal cells from samples derived from epithelial and stromal cell mixtures, only luminal cells were observed to stain positive using the antibodies against FOXA1 and GATA3, as well as ER (Figure 3D-L). Additionally there was a significant trend for early neoplasias to have a much higher fraction of cells staining positive for FOXA1 and GATA3 than normal, with positive cell fractions similar to those observed in cancer (Figure 4B,C; Table S10 in Additional file 1). This confirms that *FOXA1* and *GATA3* expression is commonly and recurrently elevated in the majority of early neoplasias (40/59 cases strong and 15/59 cases intermediate staining for FOXA1; 31/57 cases strong and 21/57 cases intermediate staining for GATA3). While the ER gene (*ESR1*) did not show significantly altered transcript levels within either the early neoplasias or breast cancers in our sequencing dataset, IHC staining for ER showed elevated protein levels in both the early neoplasias (57/61 cases with strong or intermediate staining) and breast cancers (79/99 with strong or intermediate staining; Figure 4C; Table S10 in Additional file 1). When these results were compared with IHC stains of FOXA1 and GATA3 on the same cases, there was significant association of positive FOXA1 and GATA3 cases with cases that were also positive for ER (Figure S1 in Additional file 2), suggesting an up-regulation of the ER pathway, possibly driven by FOXA1 and GATA3, may be an important early event in the majority of early neoplasias.

### Long non-coding RNAs modified in early neoplasias

lncRNAs are gaining attention as potential regulators in cancer development. When 1,376 of the previously defined cancer-expressed lncRNAs [11] were analyzed in this study, we observed similar fractions of lncRNA transcripts experiencing modified expression in early neoplasias relative to normal (4.4% lncRNAs up-regulated; 0.7% lncRNAs down-regulated; compared with 4.1% RefSeq genes up-regulated and 1.4% RefSeq genes down-regulated; Tables 1 and 2; Table S11 in Additional file 1), indicating no global enrichment for lncRNA modifications compared with RefSeq genes in this early stage of cancer development. Interestingly, fewer lncRNAs showed modifications between early neoplasia and cancer (2.0% lncRNAs up-regulated; 4.8% lncRNAs down-regulated; compared with 9.4% RefSeq genes up-regulated and 9.7% RefSeq genes down-regulated; *P* < 1 × 10^-10^; hypergeometric test; Tables 1 and 2; Table S11 in Additional file 1), indicating that fewer lncRNAs may be involved in the stages of cancer development following the early neoplasia stage. Of the numerous lncRNAs and novel transcripts up-regulated at the early neoplasia stage, the previously identified novel transcript (transcript 13741) whose transcription was specific to breast tissue and highly correlated with ER + breast cancers [11] was the highest ranking differentially up-regulated transcript in early neoplasias relative to normal within our sequencing dataset (paired SAMseq; Table S11 in Additional file 1; see Materials and methods). When visualized using RNA *in situ* hybridization, this transcript showed elevated expression in early neoplasia and cancer compared with normal (Figure 3M-O), with staining patterns correlating well with the elevated staining of ER, FOXA1, and GATA3 in the majority of neoplasia cases (data not shown). This suggests that transcript 13741 may have a role in the ER/FOXA1/GATA3 pathway being activated in early neoplasias and maintaining elevated levels within cancer.

**Table 2:** lncRNAs differentially expressed

	**lncRNAs up-regulated**	**lncRNAs down-regulated**	**Total lncRNAs differentially expressed**
Normal versus early neoplasia	61 (4.4%)	9 (0.7%)	70 (5.1%)
Normal versus cancer	118 (8.6%)	106 (7.7%)	224 (16.3%)
Early neoplasia versus cancer	27 (2.0%)	66 (4.8%)	93 (6.8%)

Paired SAMseq analysis (n = 1,376), FDR <5%. Sixteen samples per class. Numbers and percentages reported are lncRNAs up- or down-regulated in early neoplasia relative to normal, and cancer relative to either normal or early neoplasia, respectively.

### Stromal microenvironment varies in early neoplasias

The tumor microenvironment is known to play an important role in tumorigenesis. The presence of stromal cells within the tissue cores assayed by 3SEQ in this study provides the unique opportunity to examine differences of gene expression resulting from the tumor microenvironment of our samples. We identified the immunoglobulin kappa chain (*IGKC*), a B-cell immune gene gaining attention as a stromal biomarker predictive of prognosis in breast cancer and other carcinomas [20], as highly and differentially expressed within the sequencing results from our samples, decreasing in early neoplasias relative to normal tissue (Table S11 in Additional file 1). When stained using RNA *in situ* hybridization, this transcript was absent from luminal cells and specifically stained immune cells situated in the tissue stroma (Figure S2 in Additional file 2). Notably, early neoplasias showed much less staining and were associated with fewer immune cells (Figure S2 in Additional file 2 and data not shown). This example demonstrates that non-luminal cell transcription contributes to and can be detected within our 3SEQ dataset, and suggests that immune cell association with early neoplasias, or lack therefore, is a recurrent feature at this early stage whose significance will require further study.

**Figure 5:**
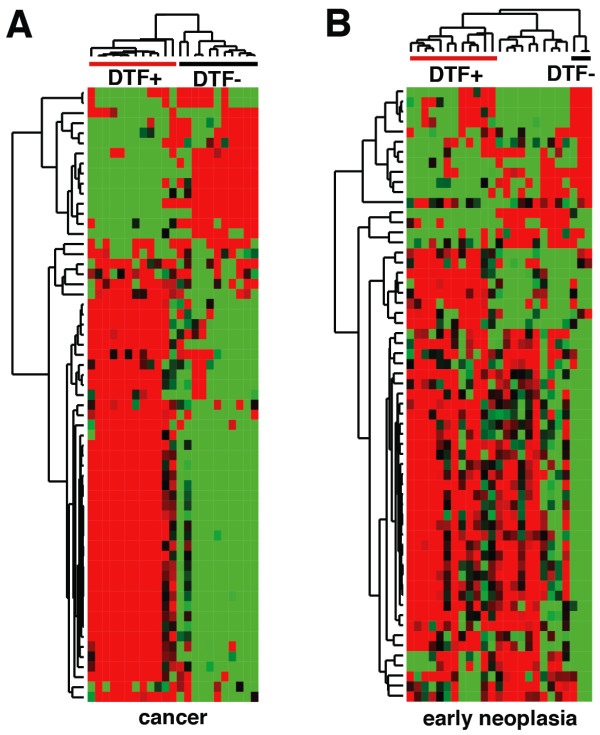
**Early neoplasias show altered DTF core gene expression. (A,B)** Heatmaps showing cancer **(A)** and early neoplasia **(B)** samples hierarchically clustered using the DTF core gene signature [20]. Samples were classified as DTF + or DFT- based on the coordinated up-regulation (red) or down-regulation (green), respectively, of approximately 40 genes.

Fibroblastic change is another stromal feature observed within breast cancer, and the stromal genes comprising the DTF gene expression signature can stratify breast cancers prognostically [21]. Here we applied the core DTF gene set to identify the DTF-positive (fibroblast response) and DTF-negative (absence of fibroblastic response) breast cancers in our analysis (Figure 5A) and compared these patterns of expression with those from the early neoplasias (Figure 5B) and normal tissue (Figure S3 in Additional file 2). Early neoplasias clustered using the core DTF gene set fell into three different groups: those showing coordinated co-expression of the DTF genes (DTF-positive; n = 12), those with an inverse DTF expression pattern as in DTF-negative cancer (n = 3), and those cases that were not clearly positive or negative (n = 10; Figure 5B). While there is a trend for patients with DTF-positive cancers to also show DTF-positive early neoplasias and normal samples, and vice versa, the correlation is not absolute (Figure 3 in Additional file 2). These observations suggest that prognostic features of the tumor microenvironment typically observed in later stage breast cancers are also present within some early neoplasia and normal samples, and its significance in the early stages of breast cancer development will need further examination.

## Discussion

The early stages of breast cancer development are poorly understood. While a number of environmental factors leading to breast cancer have been identified [3], there is a significant gap in our understanding of how these pro-cancer environmental factors function and how their influences manifest in breast cancer development. Existing genomic studies have largely focused on invasive carcinomas and metastasis, which have undergone significant genomic changes that likely include early ones that functioned in initiating oncogenesis, later ones that may confer invasive and metastatic phenotypes, as well as many genomic changes of no functional consequence. While we know which mutations, aneuploidies, and expression changes occur in carcinoma, we have little insight as to the dynamics of the genomic changes. We do not know when these changes occur, in what order they occur, and how they influence the risk of becoming cancer.

Morphologic studies have indicated that early neoplasias, such as flat epithelial atypia and ADH, and possibly less advanced early neoplasias represent direct precursors to DCIS and invasive carcinoma. The molecular profiles of early neoplasias are likely to be enriched with genetic events that initiate oncogenesis compared with molecular profiles of more advanced neoplasias like DCIS and IDC. No large-scale study of genomic alterations has been performed for neoplasia progression, and prior research on pre-invasive breast neoplasias has largely focused on DNA copy number changes [22-25], without regard for matched progression to invasive carcinoma. Our recent full-genome sequencing study of 31 samples from 6 patients, including 14 early neoplasias, provided the most complete picture to date of mutations and aneuploidies present within early neoplasias, and definitively established a genetic relationship between early neoplasias and the adjacent invasive cancer [7]. Importantly, these cancer-associated early neoplasias have already acquired aneuploidies, common within breast cancer, and hundreds of mutations, suggesting that critical oncogenic events are occurring at this early stage. Thus, some of the early neoplasias studied in this manuscript likely represent epithelium primed for critical oncogenic steps and others represent direct precursors. It is clear then that we are dealing with a mixture of cases that are related to the IDC (and are associated with progression) and cases that are not related. While this uncertainty limits the precision of this study, this feature also makes this neoplastic class interesting.

Examination of gene expression within early neoplasias is difficult, as these small lesions can only be identified under the microscope. As a result, techniques for gene expression profiling that require fresh frozen material cannot be used for most instances of early neoplasia. Previously, we demonstrated the feasibility and utility of using archival RNA from FFPE material to obtain quantitative global gene expression data using 3SEQ [10,11]. By measuring gene levels using only the 3′ ends of transcripts, we were able to quantitatively profile fragmented RNA from archival specimens. The samples surveyed within these previous studies, however, represented well-established carcinomas and sarcomas for which large amounts of input material were available for profiling. In this study we demonstrate for the first time effective 3SEQ profiling of the much smaller early neoplasia samples, for which reduced amounts of input total RNA are available. By applying 3SEQ to early neoplasias as well as patient-matched normal and cancer samples, we provide for the first time a detailed look into the global gene expression of early neoplasias, including how they differ from matched normal and cancer samples, and we shed light on the expression changes that characterize this early stage of luminal cell transformation on the progression to invasive cancer.

Our study showed that despite the genetic heterogeneity in the early neoplasias, a strong pattern of shared transcriptional modifications can be detected across early neoplasias as a whole. Expression differences can distinguish early neoplasias from both normal breast tissue and cancer, and hundreds of genes and tens of lncRNAs characterize this early stage of progression. Early neoplasias with and without adjacent cancer were indistinguishable in our analyses. Enrichment analysis using the genes modified during the progression from normal to early neoplasia suggests involvement of a number of biological processes (Table S12 in Additional file 1; see Materials and methods).

Numerous known breast cancer genes show modified transcription within the early neoplasias. Elevated levels of the *ERBB2* (HER2) transcript were unexpected in these samples because only three of the associated IDCs had HER2 amplification. While HER2 amplification has been found to occur at the ADH stage, the morphologic stage that may represent the stage just after the early neoplasias profiled here [26], amplification is not likely to explain the increased transcript levels for the majority of the early neoplasias studied here. Instead the elevated *ERBB2* transcript levels in early neoplasias suggest that HER2 may be functioning in a non-amplified setting. This is reminiscent of the recent work demonstrating the importance of elevated HER2 levels in non-amplified HER2 breast cancer stem cells [27] and suggests that HER2 may be playing a role in the early stages of cancer development, setting the stage for future oncogenic events. Our findings that ER, FOXA1, and GATA3 are all expressed at levels comparable to their matched IDC suggest that the oncogenic nature of ER pathway activation is established in the earliest stages of cancer development. Recent literature suggests that *FOXA1* may be a 'pioneer' factor necessary for establishing chromatin accessibility of ER to its target genes [28]. Therefore, *FOXA1* may be one of the important early events in ER pathway activation, setting the stage for subsequent cancer development. The role for the transcription factor GATA3 at this early stage is less clear. Work has suggested that GATA3 may act as a differentiation factor within breast cells that, when lost, typically through mutation, allows cancer progression [29]. GATA3 levels were elevated in both the early neoplasias and cancers in this study and were highly correlated with ER and FOXA1 expression, but mutation status in these samples is unknown. ER pathway activation within the early neoplasias suggests that this event alone is not sufficient for a cancer phenotype but may be important for priming cells for later events necessary for the development of cancer.

The effects of early mutations on gene expression changes were not readily discernible. Activating mutations of *PIK3CA* are present in 36% of all breast cancers, significantly enriched within luminal A breast cancers (45%) [1], and function by activating the PI3K/AKT pathway to alter a number of cellular processes, including cell proliferation, differentiation, and survival [30]. *PIK3CA* was previously detected as mutated in 13 of the 20 early neoplasias profiled within this study (Table S1 in Additional file 1) [8]. Strikingly, despite this well-known, common mutation target, we were not able to detect associated expression changes. Transcript levels of *PIK3CA* were not significantly different between mutant and wild-type early neoplasias, and these two groups did not show any of the previously described transcriptional changes found in IDC [19]. Gain of chromosome 1q is another common alteration observed in breast cancers. Our prior sequencing analysis identified this as a common aneuploidy within the early neoplasias examined [7]. Here we used FISH to identify the chromosome 1q gain in one of the cases previously sequenced as well as one other early neoplasia sample out of the 10 early neoplasia cases evaluated. While we were unable to classify these two groups based on gene expression differences, we did identify a list of 98 genes from across the genome all down-regulated in the chromosome 1q samples versus wild type. Aneuploidy work in yeast has shown that copy number alterations of chromosome arms can effect gene expression globally and is not limited to genes on the effected chromosome [31,32]. Our preliminary results here also indicate that the gain of chromosome 1q may have global effects on gene expression, and given its prevalence in early neoplasias associated with cancer, it may be important for the earliest stages of cancer development.

Previously we profiled the expression of lncRNAs within a variety of cancer types to determine global patterns of lncRNA expression in cancer [11]. lncRNAs are gaining attention for their regulatory roles within cancer. The prototype lncRNA is HOX antisense intergenic RNA (*HOTAIR*), a transcript shown to regulate HOX gene transcription and whose elevated levels in breast cancer are associated with patient outcome [33]. The search for additional lncRNA regulators with roles in cancer has led numerous studies to catalog and profile lncRNAs in a number of tissues and settings. Here we examined expression of the cancer-expressed lncRNAs within early neoplasias to identify global patterns of lncRNA usage in this early stage of cancer development. We identified numerous lncRNAs expressed within early neoplasias and differentially expressed when compared to normal tissue and cancer. Interestingly, the depletion of differentially expressed lncRNAs between early neoplasia and cancer suggests that lncRNAs may play more of a role early in the progression process, at the early neoplasia stage, and less of a role during later stages of cancer development. We had previously identified nuclear transcript 13741, along with coordinately expressed transcripts from the same 100 kb region on chromosome 10, to be highly expressed in breast cancer and correlated with ER + breast cancer [11], and others have found it associated with breast cancer recurrence [34]. Here we observed that this transcript is significantly elevated in early neoplasias compared to normal tissue using both 3SEQ and RNA *in situ* hybridization, and it shows correlated expression with the ER pathway members ER, FOXA1, and GATA3.

This study has identified a number of transcriptional features that characterize early neoplasias and provides insight into the molecular mechanisms at work during this early stage of neoplastic transformation. In our study, many of the early neoplasia samples were derived from specimens that also had the matched cancer sample. While we cannot determine relatedness based on gene expression profiling, we do have some insight into the relatedness of this class of sample from our prior whole genome sequencing of early neoplasia study [7]. By sequencing a series of patient-matched specimens similar to those in the current manuscript and comparing single nucleotide variations and validating with copy number changes, we have created evolutionary trees of the different breast neoplasia samples from normal breast to early neoplasia to DCIS to IDC. In that study, we found that 4 of 14 early neoplasias were evolutionarily related to the invasive cancer (two of these cases are represented in the current manuscript’s cohort). Thus, some of the early neoplasias studied in this manuscript likely represent epithelium primed for critical oncogenic steps and others represent direct precursors. Given the morphologic diversity of early neoplasias, their differences in association with both concurrent and future cancer, and the recently described genetic differences in 1q gain and *PIK3CA* mutation frequencies, we were struck by the similarity of their transcriptional profiles. The differences separating early neoplasias from both normal tissue and cancer were larger than any differences examined between subgroups of early neoplasias, even when corrected for smaller group sample sizes. Unsupervised clustering identified no robust subgroups of early neoplasias. Instead, the early neoplasias in our study show a common set of expression changes that appear to characterize the early neoplasias as a whole. Elevated levels of the known breast cancer genes along with a host of others are selectively modified at very early stages in the neoplastic transformation process in most cases. The events that drive further progression to cancer in a fraction of cases remain to be determined, but activation of the ER pathway early on may prime cells for additional events necessary for cancer progression. Improved characterization of these early molecular events with cancer progression with future studies will hopefully pave the way for detection and prevention tools to improve patient care.

## Conclusions

This study represents the first global examination of gene expression within early neoplasias, and identifies several features that appear to characterize early neoplasias as a whole and represent insights into this very early stage of cancer development.

## Materials and methods

### Samples

Tumor and normal samples were collected using HIPAA compliant Stanford University Medical Center institutional review board approved written informed consent. Some of the tissues already existed in tissue banks and fall under exemption 4. The FFPE tissue blocks were archived with the Stanford University and Oregon Health and Sciences University Departments of Pathology. Histopathological sections of breast resection specimens were screened for the presence of early neoplasia (specifically columnar cell change with and without atypia) by RBW. For tissue blocks possessing early neoplasia, 2-mm diameter core punches were used to collect dense areas of neoplasia and adjacent normal breast epithelial content separately. When DCIS and/or IDC were present, these were also cored. Three to ten tissue cores were pooled for each diagnosis per patient, and tissue cores were re-embedded in paraffin and re-evaluated histologically for lesion purity by sectioning length-wise along the core. Longitudinal examination of the cells across the depth of the tissue cores enabled us to observe contamination of our early neoplasia samples with normal breast epithelium or cells of any other pathology (ADH, DCIS, or IDC). Only samples that possessed >90% of luminal cells with the appropriate diagnosis within the epithelial compartment were included among our samples. Adjacent normal cores contained no identifiable pathologies, and cancer cores possessed less than 5% early neoplasia or normal epithelial cells. In all cases stromal cells were present in the core samples. DNA and total RNA were purified from paraffin slices of the embedded tissue cores using the RecoverAll Total Nucleic Acid Isolation Kit (Ambion/Life Technologies, Austin, TX, USA, catalog #1975). Briefly, deparaffination with a xylene incubation was followed by an ethanol wash and protease digestion. DNA was used for analysis of hotspot mutations and published separately [8]. Here, total RNA was used for 3SEQ library preparation.

### 3SEQ library preparation and sequencing

We optimized the 3SEQ library preparation method to work with reduced amounts of input total RNA to allow for profiling of the much smaller early neoplasia samples. 3SEQ libraries were prepared from as little as 5 μg total RNA. RNA was enriched for 3′ ends by using the Oligotex mRNA mini Kit (QIAgen, Valencia, CA, USA, catalog #70022). The 3′ poly(A)-enriched RNA pools were made into directional 3SEQ Illumina sequencing libraries, as previously described [10,11] and summarized here. RNA that was not sufficiently fragmented was heat-sheared to a size of approximately 100 to 200 bases by incubation with First Strand Buffer (Invitrogen/Life Technologies, Grand Island, NY, USA, catalog #18080-044) and oligo-dT-P7 primer (5′-CAA GCA GAA GAC GGC ATA CGA GCT CTT CCG ATC TTT TTT TTT TTT TTT TTT TTT TTT TVN-3′) at 85°C for 3 to 5 minutes, depending on the extent of fragmentation required. The mixture was cooled to 50°C and first strand cDNA synthesis was performed in a 20 μl reaction using Superscript III Reverse Transcriptase (Invitrogen/Life Technologies, catalog #18080-044) for 1 hour at 50°C. Second strand cDNA was synthesized using Second Strand Buffer (Invitrogen/Life Technologies, catalog #10812-014), DNA ligase (Invitrogen/Life Technologies, catalog #18052-019), DNA polymerase I (New England Biolabs, Ipswich, MA, USA, catalog #M0209L), and RNaseH (Epicentre Biotechnologies/Illumina, Madison, WI, USA, catalog #R0601K), and purified using the QIAquick PCR Purification Kit (QIAgen, catalog #28104). 3′ End repair and modification used dATP (Invitrogen/Life Technologies, catalog #10216018), Klenow exo (New England Biolabs, catalog #M0212L) and Klenow Buffer (NEB Buffer 2, New England Biolabs, catalog #B7002S), and was purified using the QIAgen MinElute PCR Purification Kit (QIAgen, catalog #28204). cDNA was then ligated to the double-stranded P5 linker (Adapter Oligo Mix; Illumina, San Diego, CA, USA) for 15 minutes at 22°C. SPRI bead purification (Agencourt Biosciences/Beckman Coulter, Beverly, MA, catalog #A63880) was performed both prior to and following PCR amplification of the linker-ligated cDNA. PCR reactions with 5′-AAT GAT ACG GCG ACC ACC GAG ATC TAC ACT CTT TCC CTA CAC GAC GCT CTT CCG ATC T-3′ and 5′-CAA GCA GAA GAC GGC ATA CGA GCT CTT CCG ATC-3′ primers and the Phusion PCR Master Mix (New England Biolabs, catalog #M0531) used a 15 cycle program (98°C for 30 seconds; 15 cycles of 98°C for 10 seconds, 65°C for 30 seconds, 72°C for 30 seconds; 72°C for 5 minutes). The libraries were then size selected for 200 to 300 bp fragments by agarose gel fractionation (3% Nusieve GTG; Lonza/Fisher Scientific, Pittsburgh, PA, USA) and purified using the QIAquick Gel Extraction Kit (QIAgen; catalog #28704). The libraries contain P7 sequence at the 3′ end downstream of poly(A) and P5 at the 5′ end. 3SEQ libraries were sequenced with Illumina GAIIx machines to obtain 36-base directional, single-end sequence reads. The first 25 bases of the sequence reads were mapped to hg18 with a two-mismatch allowance using ELAND (Illumina) and further filtered to remove mapping artifacts caused by ambiguous mapping, as previously described [11]. A minimum of 1.4 million uniquely mapping reads were obtained for each library (Table S2 in Additional file 1). Reads within exons of RefSeq genes (n = 22,775 genes; downloaded July 2011 from the University of California Santa Cruz Genome browser) were tallied to obtain the total number of reads per gene for each sample (see Additional file 3 for scripts). See Gene Expression Omnibus accession [GEO:GSE47462] for raw fastq files and the file of raw gene counts.

### RNAseq library preparation and sequencing

Samples were obtained using methods identical to the original cohort. However, for this validation cohort we used full transcriptome RNAseq, not 3SEQ, with rRNA depleted non-polyA selected transcriptome library. Total RNA was extracted and purified from FFPE materials with a commercially available kit (Recover All Total Nucleic Acid Isolation Kit; Ambion, catalog #AM1975).

Both cytoplasmic (nuclear-encoded) and mitochondrial rRNA were depleted using the Ribo-Zero Magnetic Human Gold Kit (Epicentre, Madison, WI, an Illumina Company; catalog #MRZG12324). Input total RNA (1 to 4 μg) was incubated with removal solution containing specific probes according to instructions and incubated at 68°C for 10 minutes. Cytoplasmic and rRNA bound to probes were removed by magnetic bead pull-down. The final ribosomal-depleted RNA was recovered after sodium acetate/glycogen addition and ethanol precipitation overnight. Samples were centrifuged at 10,000 g for 30 minutes, washed once per instruction, and resuspended with 17 μl of EPF (Elute, Prime, Fragment Mix, from the kit of TruSeq RNA sample Preparation v2, Illumina; catalog #RS-122-2001). RNA was fragmented at 94°C for 15 seconds. The remaining library construction was performed as above. The libraries were quantified with Qubit 2.0 Fluoro meter (Life Technologies, Foster City, CA, USA) and validated with BioAnalyzer 2100 (Agilent).

Libraries were sequenced with Illumina HiSeq machines to obtain 101-base directional, paired-end sequence reads. These 101 bp paired-end reads were uniquely mapped to hg19 using Bowtie2/TopHat2 with the default settings [35]. Duplicates were removed using Picard. Fragments per kilobase of exon per million fragments mapped (FPKM) for gene expression was calculated using Cufflinks v2.1.1 [36] (see Additional file 4).

### Classification analysis

Genes with an average of less than one read per sample (<72 reads across all samples) were removed from the dataset, resulting in a dataset of 15,748 (≥72 reads across the samples) genes that was then normalized and transformed using package SAM 2.0 [13]. Briefly, the counts from the data were transformed using Anscombe transformation [37] to stabilize variance, and then normalized using sequencing depths estimated by PoissonSeq [38]. The resulting normalized data are roughly Gaussian-distributed and the values are comparable across samples [12], resembling microarray data. This dataset was used to perform a three-class (unpaired: normal, early neoplasia, cancer) PAM analysis [12] using 10-fold cross-validation on the 72 samples. Briefly, PAM shrinks the centroids of gene expression of each class to their overall centroid and classifies the samples by the nearest centroid rule. The classifier only uses a sparse set of genes whose class-specific centroids are different from the overall centroid after shrinkage, and the size of the sparse set is chosen by minimizing the cross-validation error. DCIS and invasive cancers were combined into the 'cancer' class for this study. A two class (unpaired) PAM analysis was also performed using 10-fold cross-validation on the 49 normal and early neoplasia samples (see Additional file 3 for scripts). Validation of using the TCGA RNAseq data from breast invasive carcinoma samples and the matched normal samples utilized the level 3 data from batch 93. This dataset contains expression levels measured by RNASeqV2 of 20,502 genes in 45 breast invasive carcinoma samples and 16 matched normal samples. We filtered 2,212 genes as they have too few numbers of reads across samples. We then used SAMseq to detect significant genes and estimate the adjusted *P*-values (FDR).

### Differentially expressed genes

To leverage the patient-matched samples in this study, we examined data from the subset of patients possessing samples of all three diagnostic classes: normal, early neoplasia, and cancer (DCIS or invasive). Right and left breast samples obtained from one patient were treated as independent cases. For the three patients who had more than one sample for a diagnostic class (two cancer samples, for example), we used the sample with the larger sequencing depth. In this way, we developed a truncated dataset with a single instance of matched normal, early neoplasia, and cancer for each of 16 patients. This dataset was filtered to remove genes with fewer than five reads in each diagnostic class, and normalized using SAM 2.0 as described above, to yield a dataset of 13,643 genes for further analysis. Paired differential expression analysis was performed using SAMseq [13]. Briefly, count data are stochastically normalized sequencing depths using 'Poisson resampling' [13], and nonparametric statistics are calculated to measure the expression differences among classes. Next, permutations are performed to obtain the null distribution of these nonparametric statistics. It has been shown that compared with parametric methods (often based on negative-binomial distribution), SAMseq is more powerful and robust to noise when the sample size is moderate or large [39]. Differentially expressed genes at FDR <5% were identified and are reported in Tables S5 to S8 in Additional file 1 (see Additional file 3 for scripts).

### Fluorescence *in situ* hybridization

FISH was performed to examine chromosome 1q gain using 4-μm FFPE sections from 10 of the early neoplasia samples (Table S8 in Additional file 1). Bacterial artificial chromosome (BAC) clones RP11-1044H13 (1q32) and RP11-1120 M18 (3q25) were obtained from the BACPAC Resources Centre (Children’s Hospital Oakland Research Institute, Oakland, CA, USA), while clone CTD-2344 F21 (2q37) was from Invitrogen/Life Technologies. Probe RP11-1044H13 was labeled with Cy3 dUTP (Amersham/GE Healthcare, Pittsburgh, PA, USA) and control probes RP11-1120 M18 and CTD-2344 F21 were labeled with AlexaFluor 647-aha-dUTP (Invitrogen/Life Technologies, catalog #A32763) and Green dUTP (Abbot Molecular, Des Plaines, IL, USA; catalog #02 N32-050), respectively, using the Nick Translation Kit (Abbot Molecular). Slides were deparaffinized in xylene twice for 10 minutes, dehydrated twice with 100% ethanol, air dried for 10 minutes, and then pretreated in 10 mM citric acid pH6 at 80°C for 45 minutes. Slides were digested for 40 minutes in pepsin (75,000 units; Sigma-Aldrich, St Louis, MO, USA; catalog #P6887) at 37°C. Fluorescent labeled probes and slides were co-denatured at 75°C for 7 minutes and hybridized at 37°C for 16 to 18 hours in a humidified chamber. Post-hybridization washes were performed using 2×SSC/0.3% NP-40 at 72°C for 5 minutes. Slides were dehydrated and air dried in the dark, and counterstained with DAPI (Invitrogen/Life Technologies, catalog #P36935). Imaging and analysis were performed using Ariol 3.4v software (Genetix/Leica Microsystems, Wetzlar, Germany). Fluorescence was scored visually using filters for 550 nm (green, Cy3), 647 nm (red, AlexaFluor 647), and 488 nm (yellow, Abbot Green). Total signals for each color within a given slide region were counted, regardless of nucleus. Signals from roughly 40 to 300 cells per sample were counted. Total green counts (1q32) were compared with red (3q25 control) and yellow (2q37 control) counts. Those samples with ratios 1.5 or greater were considered amplified for 1q (Table S8 in Additional file 1).

### Immunohistochemistry

Primary antibodies were directed toward FOXA1 (NBP1-49791, rabbit polyclonal, Novus Biologicals, Littleton, CO, USA), GATA3 (HG3-35, mouse monoclonal, Santa Cruz Biotechnology, Dallas, TX, USA), and ER (SP1, rabbit monoclonal, Abcam, Cambridge, MA, USA). Tissue microarrays (Stanford TA375, TA386, TA387, TA390, TA392, TA393) were constructed using a technique previously described [40] with a tissue arrayer (Beecher Instruments, Silver Spring, MD, USA). We took 615 cores (0.6 or 2.0 mm) from paraffin-embedded breast samples from 105 patients with early neoplasia archived with the Stanford University Medical Center between 2000 and 2012. Multiple cores were taken for each patient. Cores often possessed more than one diagnosis, so total instances scored across the 615 cores represent: 105 normal, 138 early neoplasia, 161 DCIS, and 84 IDC. ER and GATA3 IHC was performed on 4 μm sections using the Ventana BenchMark XT automated immunostaining platform (Ventana Medical Systems/Roche, Tucson, AZ, USA). For FOXA1 IHC, sections of 4 μm were cut from the tissue array blocks, deparaffinized in xylene, hydrated in a graded series of alcohol, and prepared for staining using Target Retrieval Solution, Citrate pH6 (Dako/Agilent, Carpinteria, CA, USA, catalog #S2369) to retrieve antigenic sites at 116°C for 3 minutes. Staining was then performed using the EnVision + anti-rabbit system (Dako/Agilent, catalog #K4011). Estimates of the fraction of cells staining positive within the nucleus of luminal cells in the epithelial compartment were made and scores were assigned as follows: FOXA1 '0', <50% cells; FOXA1 '1', 50 to 90% cells; FOXA1 '2', >90% cells. ER and GATA3 '0', <20% cells; ER and GATA3 '1', 20 to 90% cells; ER and GATA3 '2', >90% cells. When multiple scores were obtained for a given diagnosis within a patient, the median score was used as the representative score. In this way we obtained 58 independent instances of normal, 61 early neoplasias, 91 cancer (or 88 DCIS, 51 IDC when considered separately). Many of these represented complete patient-matched sets (Table S10 in Additional file 1).

### lncRNA analysis

The peaks previously identified for 1,065 known lncRNAs and 1,071 novel transcripts were profiled by counting sequencing reads falling within the previously defined 3SEQ peak coordinates [11]. A dataset of 48 samples comprising the 16 normal, early neoplasia, and cancer sets of patient-matched samples was filtered to remove transcripts with fewer than five reads in at least one diagnostic class, yielding 1,376 transcripts for further analysis. A series of paired SAMseq analyses was performed using the SAM 2.0 R package as described above [12]. Differentially expressed genes at FDR <5% were identified and are reported in Table S10 in Additional file 1 (see Additional file 3 for scripts).

### RNA *in situ* hybridization

The RNA *in situ* hybridization probe for *IGKC* was designed against chr2: 88,937,790-88,938,290 (hg18) using primer 5′-CTG TTG TGT GCC TGC TGA AT-3′ and the T7 promoter-tagged primer 5′-CTA ATA CGA CTC ACT ATA GGG TTA AAG CCA AGG AGG AGG AG-3′. RNA *in situ* hybridizations for transcript 13741 (probe described previously [11]) and *IGKC* were performed on TA375, as described previously [11].

### DTF core gene analysis

Normalized expression data for 61 of the 66 genes from the DTF core gene signature [20] were used to hierarchically cluster the cancer, early neoplasia, and normal samples separately, using average linkage clustering of centered data with Cluster 3.0. Heatmaps were visualized using JavaTreeView. Samples were classified as DTF+, DFT-, or undetermined based on coordinated up-regulation of approximately 40 genes, as seen in Figure 5 and Figure S3 in Additional file 2.

## Abbreviations

3SEQ: 3′-end sequencing for expression quantification; ADH: atypical ductal hyperplasia; DCIS: ductal carcinoma *in situ*; ER: estrogen receptor; FDR: false discovery rate; FFPE: formalin-fixed paraffin-embedded; FISH: fluorescence *in situ* hybridization; IDC: invasive ductal carcinoma; IHC: immunohistochemistry; lncRNA: long non-coding RNA; PAM: Prediction Analysis of Microarrays; SAM: Significance Analysis of Microarrays; TCGA: The Cancer Genome Atlas.

## Competing interests

The authors declare that they have no competing interests.

## Authors’ contributions

ALB, JL, and RBW designed the study, analyzed the data, and wrote the paper. XG and SV built tissue microarrays. RTS analyzed the RNAseq data. SV performed the immunohistochemistry and fluorescence *in situ* hybridization analyses. SXZ extracted RNA, and optimized and built 3SEQ and RNAseq libraries. RL performed the RNA *in situ* hybridization analyses. RT provided statistical guidance. All authors read and approved the final manuscript.

## Authors’ information

Alayne L Brunner and Jun Li are co-first authors.

## Supplementary Material

Additional file 1: Table S1 contains a summary of the samples, including clinical and *PIK3CA* mutation status. **Table S2.** lists sequencing read counts and statistics for the 3SEQ libraries. **Table S3.** shows PAM analysis cross-validated probabilities for each sample. **Table S4.** shows classification genes identified by PAM analysis. **Table S5.** shows genes differentially expressed between normal and cancer, FDR <5%. **Table S6.** shows genes differentially expressed between early neoplasia and cancer, FDR <5%. **Table S7.** shows genes differentially expressed between normal and early neoplasia, FDR <5%. The table includes information for which genes overlap genes differentially expressed in cancer, PAM classification genes, TCGA genes, Cancer Gene Census genes, and genes comprising the intrinsic breast cancer gene signature, PAM50 gene signature, and Genes-to-Systems Breast Cancer database. Normalized expression values for each of the 72 profiled samples are included. **Table S8.** shows FISH signal counts used in determining chromosome 1q gain in early neoplasias. **Table S9.** shows genes differentially expressed between early neoplasias with chromosome 1q gain and wild-type early neoplasias, FDR <5%. **Table S10.** shows immunohistochemistry scores for ER, FOXA1, and GATA3 stains. **Table S11.** shows differentially expressed lncRNAs and novel transcripts between normal tissue and early neoplasia, FDR <5%. **Table S12.** shows KEGG and PATHNER enrichment values for normal versus neoplasia genes. **Table S13.** shows significant genes between normal versus cancer for the RNAseq validation dataset based on *t*-test with a *P*-value cutoff of 0.05. **Table S14.** shows significant genes between early neoplasia versus cancer for the RNAseq validation dataset based on a *t*-test with a *P*-value cutoff of 0.05. **Table S15.** shows significant genes between normal versus early neoplasia for the RNAseq validation dataset based on a *t*-test with a *P*-value cutoff of 0.05. Click here for file

Additional file 2: Figure S1 plots FOXA1 and GATA3 IHC scores in relation to patient-matched ER scores. **Figure S2.** shows *IGKC* RNA *in situ* hybridization in normal tissue and early neoplasia. **Figure S3.** shows clustered heatmaps of DTF gene signatures for normal tissue, early neoplasias, and cancer, and includes gene and sample names. Click here for file

Additional file 3 **Perl and R scripts used in analysis.** See the documentation files for details. Click here for file

Additional file 4 Validation dataset for classification analysis. Click here for file
