# Supplementary material for: A shared transcriptional program in early breast neoplasias despite genetic and clinical distinctions
Source: Genome Biol. 2014 May 23;15(5):R71. doi: 10.1186/gb-2014-15-5-r71 (PMC4072957; doi:10.1186/gb-2014-15-5-r71)
Supplement: Additional file 2: Figure S1 — plots FOXA1 and GATA3 IHC scores in relation to patient-matched ER scores. Figure S2. shows IGKC RNA in situ hybridization in normal tissue and early neoplasia. Figure S3. shows clustered heatmaps of DTF gene signatures for normal tissue, early neoplasias, and cancer, and includes gene and sample names. [file gb-2014-15-5-r71-S2.docx]

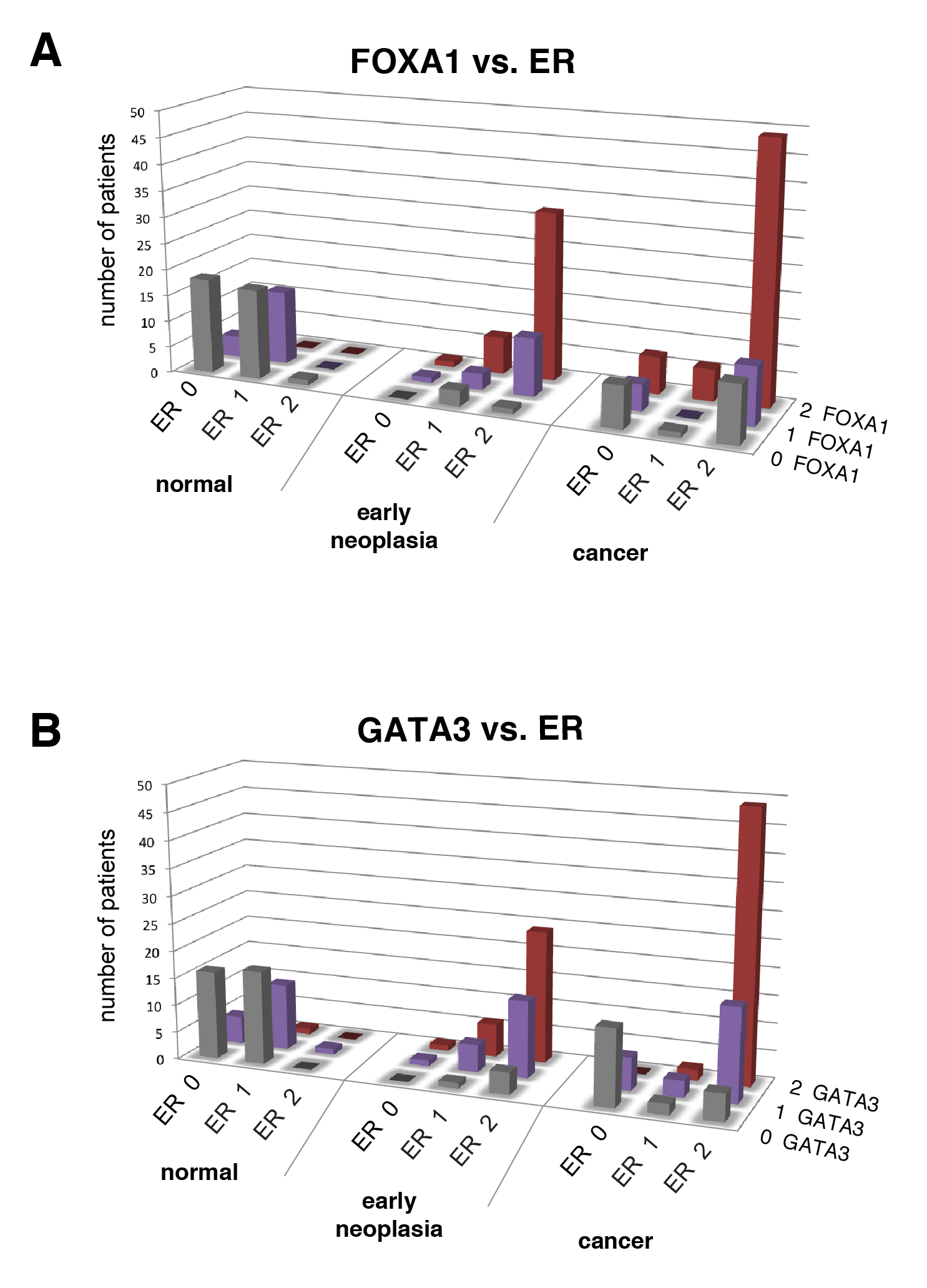


## Figure S1 - FOXA1 and GATA3 expression is correlated with ER

# Patient-matched A) FOXA1 and B) GATA3 median IHC expression scores are plotted relative to ER IHC expression scores. Scores represent the fraction of cells stained, as described in the Methods. The value of the hidden bar in both plots is 0.


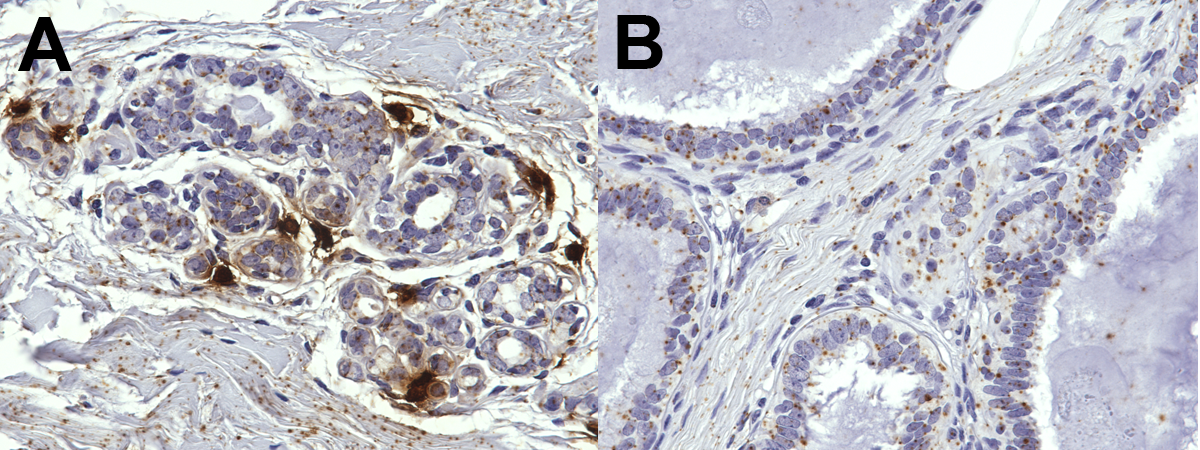


## Figure S2 - IGKC RNA *in situ* hybridization differs between normal and early neoplasia

# Representative ISH staining for IGKC on A) normal and B) early neoplasia. The presence of immune cells is highlighted by strong staining in A). Early neoplasias show much less staining and are associated with fewer immune cells, as observed in B).


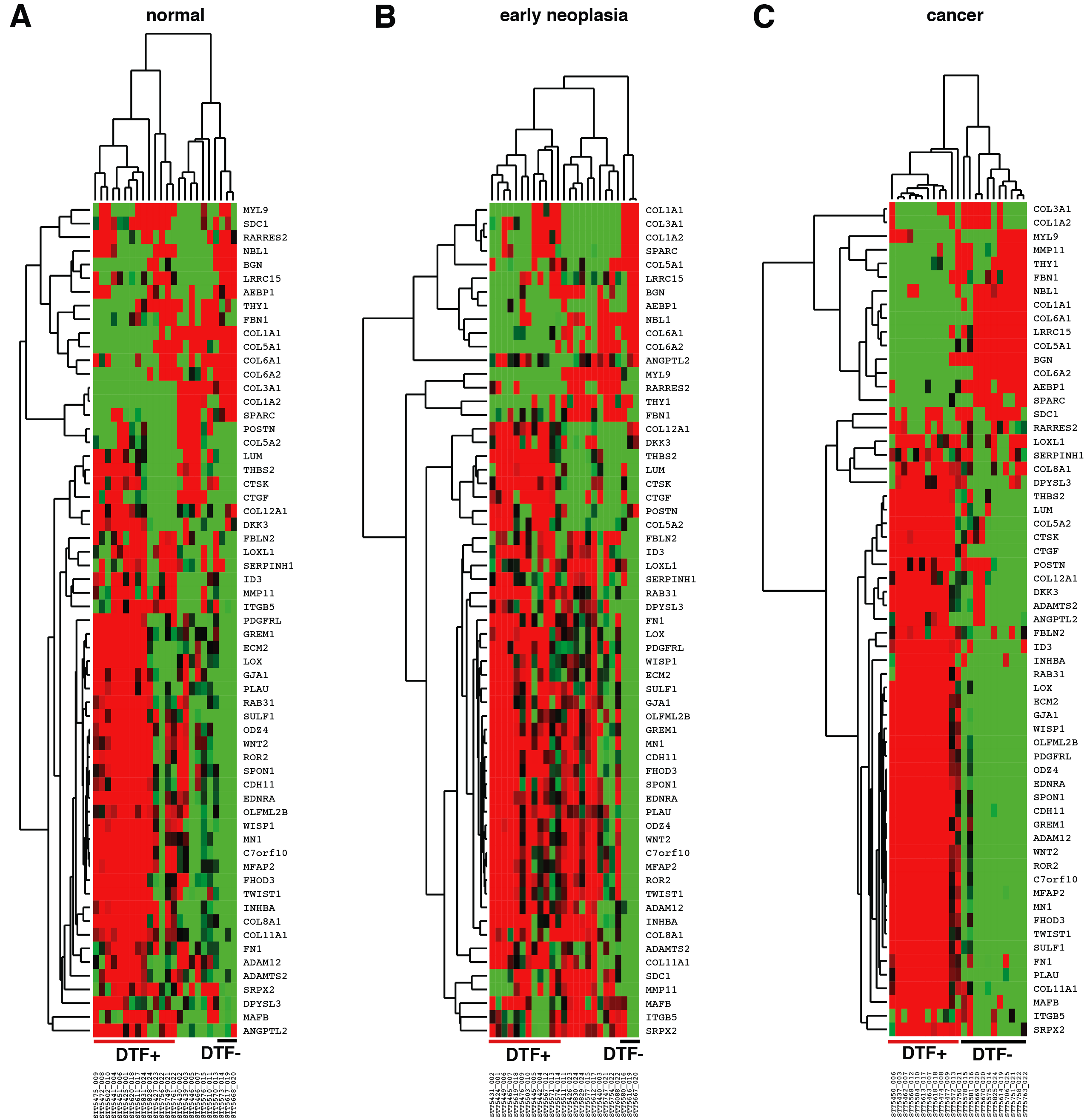


## Figure S3 - DTF gene signature present in early neoplasia and normal

# A) Normal, B) early neoplasia, and C) cancer samples clustered using the DTF core gene signature. Early neoplasia and normal samples were labeled DTF+ or DTF- by comparison with the DTF+ and DTF- cancers.
