# Supplementary material for: A shared transcriptional program in early breast neoplasias despite genetic and clinical distinctions
Source: Genome Biol. 2014 May 23;15(5):R71. doi: 10.1186/gb-2014-15-5-r71 (PMC4072957; doi:10.1186/gb-2014-15-5-r71)
Supplement: Additional file 3 — Perl and R scripts used in analysis. See the documentation files for details. [file gb-2014-15-5-r71-S3.zip › Additional_file_3/Additional file 3.docx]

## Additional file 3 – Scripts used in analysis

**Processing read files to obtain gene counts**

1) Bed files possessing mapped sequence reads were overlapped with a custom bed file containing coordinates for all RefSeq exons using BedTools (Version 2.12.0 April-3-2011).

Example line of read-containing bed file:

## chr7 44120603 44120604 TAAGAGATGGGGTTGCTGCAGTGTT 0 + 0 0 0,0,255

Example line of exon bed file (exon_annotation_file_hg18_symbol_110901.bed):

## chr1 4224 4692 WASH7P 0 -

Command used to perform overlap:

> intersectBed -a 080606_GA-EAS46_0006_20JDH_L4.bed -b exon_annotation_file_hg18_symbol_110901.bed -wa -wb -s > 080606_GA-EAS46_0006_20JDH_L4.txt

Example line of output file:

## chr7 44120603 44120604 TAAGAGATGGGGTTGCTGCAGTGTT

0 + 0 0 0,0,255 chr7 44119717 44120684 AEBP1 0 +

2) Run perl script (process_overlap_file.pl) to process the output file:

> perl process_overlap_file.pl 080606_GA-EAS46_0006_20JDH_L4.txt

This script evaluates the overlap results for each read and reports all reads that overlap with a single gene symbol. (Reads that overlap more than one gene symbol are reported in the “discrepancy file” and not used in this analysis.)

Example line from “unique” output file:

## chr7 44120603 + AEBP1

3) Run perl script (count_unique_file.pl) to process reads overlapping single genes:

> perl count_unique_file.pl 080606_GA-EAS46_0006_20JDH_L4_unique.txt

This script counts the number of reads overlapping each gene symbol in “gene_file.txt”. The output file produced is a list of counts for each gene. The “gene_file.txt” was concatentated with the count file from each sample to produce the matrix table of reads per gene per sample.

**Classification analysis**

source(‘run.all.R’)

This includes two parts:

1) Read in and normalize the data for PAMR to use. This is implemented in arrange.data.R. (Output: dat.triplet.RData and dat.lnc.triplet.RData).

2) Run PAMR. This is implemented in all.classification.R.

**SAMseq analysis for differential expression**

Run 6 SAMR analyses:

RefSeq_neoplasia_cancer_SAM_paired.R

RefSeq_normal_cancer_SAM_paired.R

RefSeq_normal_neoplasia_SAM_paired.R

lncRNA_neoplasia_cancer_SAM_paired.R

lncRNA_normal_cancer_SAM_paired.R

lncRNA_normal_neoplasia_SAM_paired.R

Data is loaded from dat.triplet.RData and dat.lnc.triplet.RData.
